# The cervical cancer divide: state variation in incidence, mortality, and progress toward elimination in the United States

**DOI:** 10.1093/jncics/pkag005

**Published:** 2026-01-25

**Authors:** Trisha L Amboree, Poria Dorali, Haluk Damgacioglu, Jane R Montealegre, Marvella E Ford, Brian Orr, Gweneth Lazenby, Britton Gibson, Ana P Ortiz, Tonatiuh Suárez Ramos, Kalyani Sonawane, Ashish A Deshmukh

**Affiliations:** Department of Public Health Sciences, Medical University of South Carolina, Charleston, SC, United States; Cancer Prevention and Control Program, Hollings Cancer Center, Medical University of South Carolina, Charleston, SC, United States; Department of Public Health Sciences, Medical University of South Carolina, Charleston, SC, United States; Cancer Prevention and Control Program, Hollings Cancer Center, Medical University of South Carolina, Charleston, SC, United States; Department of Public Health Sciences, Medical University of South Carolina, Charleston, SC, United States; Cancer Prevention and Control Program, Hollings Cancer Center, Medical University of South Carolina, Charleston, SC, United States; Department of Behavioral Science, The University of Texas MD Anderson Cancer Center, Houston, TX, United States; Department of Public Health Sciences, Medical University of South Carolina, Charleston, SC, United States; Cancer Prevention and Control Program, Hollings Cancer Center, Medical University of South Carolina, Charleston, SC, United States; Division of Gynecologic Oncology, Medical University of South Carolina, Hollings Cancer Center, Charleston, SC, United States; Department of Obstetrics and Gynecology, Medical University of South Carolina, Charleston, SC, United States; Department of Obstetrics and Gynecology, Medical University of South Carolina, Charleston, SC, United States; Division of Cancer Control and Population Sciences, University of Puerto Rico Comprehensive Cancer Center, San Juan, PR, United States; Division of Cancer Control and Population Sciences, University of Puerto Rico Comprehensive Cancer Center, San Juan, PR, United States; Department of Public Health Sciences, Medical University of South Carolina, Charleston, SC, United States; Cancer Prevention and Control Program, Hollings Cancer Center, Medical University of South Carolina, Charleston, SC, United States; Department of Public Health Sciences, Medical University of South Carolina, Charleston, SC, United States; Cancer Prevention and Control Program, Hollings Cancer Center, Medical University of South Carolina, Charleston, SC, United States

## Abstract

Cervical cancer elimination (<4 cases per 100 000) is a critical cancer prevention goal in the United States. Implementation of health policies and allocation of health resources occur at regional and state levels; therefore, understanding region- and state-specific cervical cancer incidence, mortality, and progress toward elimination—and remaining gaps—is essential. We estimated hysterectomy-corrected cervical cancer incidence, mortality, and progress toward elimination across all 50 states, the District of Columbia, and Puerto Rico. In 2021, Massachusetts was the only state nearing (4.3 per 100 000) the elimination threshold. Southeastern and Southwestern states were furthest, with the highest incidence rates in Mississippi (14.8), Louisiana (14.2), and Oklahoma (13.8). The mortality rate ranged from 6.8 (Alabama) to 1.4 (Wisconsin). In most states, cervical cancer incidence and mortality did not change from 2007-2011 to 2017-2021. Identifying and addressing regional- and state-level barriers impeding progress will be key to achieving cervical cancer elimination.

In 2020, the World Health Organization (WHO) launched a global strategy to eliminate cervical cancer as a public health problem, defined as achieving an annual incidence rate of fewer than 4 cases per 100 000 women per year.^1^ In the United States (US), cervical cancer elimination efforts focus on enhancing primary prevention through human papillomavirus (HPV) vaccination and secondary prevention through cervical cancer screening, early diagnosis, and treatment of precancerous lesions. Historically, cervical cancer screening has been a highly effective prevention strategy. The widespread implementation of population-based cervical cancer screening programs contributed to a substantial decline (5% per year) in cervical cancer incidence in the US from the early 1980s through the early 2000s.^2^ However, the decline has slowed considerably in the past 2 decades, averaging 1.2% per year between 2001 and 2019.^3^ In 2019, the hysterectomy-corrected cervical cancer incidence rate remained more than double (at 9.8 per 100 000) the elimination target.^3^

While national trends in incidence and mortality rates are well characterized, less is known about how these patterns vary across states, and how close individual states are to reaching the elimination target. Given that cancer prevention policies and programs are implemented at the state and regional levels, a granular understanding of cervical cancer incidence and mortality by state and region is essential to identify where progress has been made, where gaps remain, and where more intensified efforts may be needed to achieve elimination.

We used the National Program of Cancer Registries (NPCR) and the Surveillance, Epidemiology, and End Results (SEER) databases^4^ to identify microscopically-confirmed cervical cancer cases using International Classification of Diseases for Oncology, Third Edition (ICD-O-3) site codes C53.0 to C53.9 and histology codes 8010-8671 and 8940-8941, as defined by national reports and surveillance data to indicate HPV-associated cervical carcinoma.^5^ State-level cervical cancer mortality data were obtained from the National Center for Health Statistics (NCHS).^6^ Incidence and mortality rates (per 100 000 women) were estimated and age-standardized to the 2000 US standard population for the 50 states, the District of Columbia (DC), Puerto Rico, and by regions (Midwest, Non-Contiguous, Northeast, Pacific, Rocky Mountain, Southeast, and Southwest). For robust population-level estimates (consistent with US national reports^7^^,^^8^) and to support age standardization, we included all 19 age groups, starting at age 0 and grouped into 5-year categories. To account for differences in the population at risk, all rates were corrected for hysterectomy prevalence. State-specific hysterectomy prevalence was estimated using survey-weighted data from the Behavioral Risk Factor Surveillance System,^9^ which provides robust estimates of hysterectomy prevalence for each state,^10^^,^^11^ with smoothed estimates stratified by age and year. The proportion of women with a hysterectomy was removed from the population denominator. We compared rates in 2007-2011 (baseline [first period after introduction of HPV vaccination]) to contemporary (2017-2021) years by calculating rate ratios (RRs) and 95% confidence intervals (CIs). We assumed a normal distribution for log-transformed rates and excluded data from 2020 due to potential disruptions in reporting caused by the COVID-19 pandemic. HPV vaccination data from the 2023 National Immunization Survey were used to contextualize state-level primary prevention efforts.^12^ All analyses were conducted using the SEER*STAT software program (version 8.4.5),^13^ R,^14^ and SAS version 9.4.^15^

In 2021, 12 038 new cases of cervical cancer and 4351 cervical cancer deaths were reported nationwide. The Southeast and Southwest regions together accounted for 45.5% of all cervical cancer cases and 48.2% of cervical cancer deaths, with the Southeast alone contributing 30.7% of cases and 33.7% of deaths (Table 1). Nationally, the g 8.9 (95% CI = 8.9 to 9.0) and 3.2 (95% CI = 3.1 to 3.3) per 100 000 women, respectively. The Southwest had the highest regional rates, with an incidence of 11.2 (95% CI = 10.7 to 11.8) and mortality of 4.0 (95% CI = 3.7 to 4.4), whereas the Northeast had the lowest, with an incidence of 7.3 (95% CI = 7.0 to 7.7) and mortality of 2.2 (95% CI = 2.0 to 2.4).

**Table 1.** Hysterectomy-corrected cervical cancer incidence and mortality in 2021, by US region and state.

	Incidence			Mortality		
	Cases, *n*	Proportion of all cases, %	Age-standardized rate (per 100 000, 95% CI^a^)	Deaths, *n*	Proportion of all deaths, %	Age-standardized rate (per 100 000, 95% CI^a^)
**US**	12 038	100.0%	**8.9 (8.9 to 9.0)**	4351	100.0%	**3.2 (3.1 to 3.3)**
**Southwest**	1784	14.8%	**11.2 (10.7 to 11.8)**	631	14.5%	**4.0 (3.7 to 4.4)**
Oklahoma	192	1.6%	13.8 (11.9 to 15.9)	73	1.7%	5.5 (4.3 to 7.0)
Texas	1293	10.7%	12.0 (11.3 to 12.6)	434	10.0%	4.2 (3.8 to 4.6)
New Mexico	78	0.6%	9.1 (7.2 to 11.4)	24	0.6%	2.6 (1.7 to 4.0)
Arizona	221	1.8%	7.9 (6.9 to 9.0)	100	2.3%	3.5 (2.8 to 4.2)
**Southeast**	3,692	30.7%	**9.9 (9.6 to 10.2)**	1466	33.7%	**3.9 (3.7 to 4.1)**
Mississippi	152	1.3%	14.8 (12.5 to 17.4)	64	1.5%	6.3 (4.8 to 8.0)
Louisiana	225	1.9%	14.2 (12.4 to 16.2)	68	1.6%	4.5 (3.5 to 5.7)
Kentucky	219	1.8%	12.8 (11.1 to 14.7)	83	1.9%	4.8 (3.8 to 5.9)
Arkansas	135	1.1%	12.7 (10.6 to 15.1)	54	1.2%	5.2 (3.9 to 6.8)
West Virginia	84	0.7%	11.9 (9.5 to 14.8)	31	0.7%	4.0 (2.7 to 5.7)
Alabama	201	1.7%	11.5 (9.9 to 13.2)	114	2.6%	6.8 (5.6 to 8.2)
District of Columbia	32	0.3%	11.0 (7.4 to 15.6)	–	–	–
Florida	986	8.2%	10.7 (10.0 to 11.4)	372	8.5%	3.7 (3.3 to 4.1)
Georgia	408	3.4%	9.8 (8.9 to 10.8)	142	3.3%	3.6 (3.0 to 4.3)
Tennessee	238	2.0%	9.2 (8.0 to 10.4)	109	2.5%	4.3 (3.5 to 5.2)
Delaware	41	0.3%	9.0 (6.4 to 12.4)	14	0.3%	2.9 (1.6 to 5.0)
South Carolina	179	1.5%	8.9 (7.6 to 10.3)	105	2.4%	5.4 (4.4 to 6.6)
North Carolina	362	3.0%	8.5 (7.6 to 9.4)	142	3.3%	3.3 (2.7 to 3.9)
Virginia	245	2.0%	6.8 (5.9 to 7.7)	96	2.2%	2.7 (2.1 to 3.3)
Maryland	185	1.5%	6.7 (5.7 to 7.7)	72	1.7%	2.6 (2.0 to 3.2)
**Non-Contiguous**	256	2.1%	**10.3 (9.1 to 11.7)**	74	1.7%	**3.1 (2.4 to 4.0)**
Puerto Rico	189	1.6%	12.4 (10.7 to 14.4)	52	1.2%	3.1 (2.3 to 4.1)
Alaska	25	0.2%	9.2 (5.9 to 13.3)	–	–	–
Hawaii	42	0.3%	6.3 (4.5 to 8.6)	22	0.5%	3.2 (2.0 to 4.9)
**Midwest**	2131	17.7%	**8.4 (8.0 to 8.7)**	866	19.9%	**3.0 (2.8 to 3.3)**
Kansas	114	0.9%	10.3 (8.4 to 12.3)	43	1.0%	4.1 (2.9 to 5.5)
Missouri	250	2.1%	10.3 (9.1 to 11.7)	95	2.2%	3.8 (3.0 to 4.6)
Ohio	452	3.8%	9.4 (8.5 to 10.3)	156	3.6%	3.2 (2.7 to 3.8)
Iowa	111	0.9%	8.9 (7.3 to 10.8)	24	0.6%	1.7 (1.1 to 2.5)
Nebraska	64	0.5%	8.8 (6.7 to 11.2)	18	0.4%	2.3 (1.4 to 3.7)
Illinois	469	3.9%	8.5 (7.7 to 9.3)	169	3.9%	3.0 (2.6 to 3.5)
South Dakota	26	0.2%	7.5 (4.8 to 11.1)	14	0.3%	4.2 (2.3 to 7.1)
Michigan	300	2.5%	7.3 (6.5 to 8.2)	141	3.2%	3.3 (2.8 to 3.9)
North Dakota	20	0.2%	7.0 (4.2 to 10.8)	–	–	–
Minnesota	165	1.4%	6.8 (5.8 to 8.0)	54	1.2%	2.2 (1.7 to 2.9)
Wisconsin	160	1.3%	6.6 (5.6 to 7.7)	37	0.9%	1.4 (1.0 to 2.0)
Indiana^b^	–	–	–	115	2.6%	4.3 (3.6 to 5.2)
**Rocky Mountain**	501	4.2%	**8.3 (7.6 to 9.1)**	148	3.4%	**2.9 (2.4 to 3.4)**
Nevada	121	1.0%	9.8 (8.1 to 11.8)	49	1.1%	3.9 (2.9 to 5.2)
Utah	99	0.8%	9.2 (7.5 to 11.3)	29	0.7%	3.0 (2.0 to 4.3)
Wyoming	18	0.1%	8.8 (5.2 to 14.0)	–	–	–
Montana	37	0.3%	8.4 (5.9 to 11.7)	–	–	–
Colorado	177	1.5%	7.5 (6.4 to 8.7)	52	1.2%	2.2 (1.6 to 2.9)
Idaho	49	0.4%	7.1 (5.2 to 9.4)	18	0.4%	3.0 (1.8 to 4.7)
**Pacific**	1736	14.4%	**8.1 (7.7 to 8.5)**	578	13.3%	**2.6 (2.4 to 2.9)**
California	1358	11.3%	8.2 (7.8 to 8.6)	459	10.5%	2.7 (2.5 to 3.0)
Washington	248	2.1%	8.0 (7.0 to 9.1)	71	1.6%	2.1 (1.7 to 2.7)
Oregon	130	1.1%	7.4 (6.1 to 8.8)	48	1.1%	2.7 (2.0 to 3.6)
**Northeast**	1938	16.1%	**7.3 (7.0 to 7.7)**	588	13.5%	**2.2 (2.0 to 2.4)**
New Jersey	367	3.0%	8.3 (7.5 to 9.2)	105	2.4%	2.2 (1.8 to 2.7)
Maine	47	0.4%	8.2 (5.9 to 11.0)	15	0.3%	2.2 (1.2 to 3.7)
Pennsylvania	455	3.8%	8.0 (7.3 to 8.8)	155	3.6%	2.6 (2.2 to 3.1)
New York	742	6.2%	7.9 (7.3 to 8.5)	216	5.0%	2.2 (1.9 to 2.5)
New Hampshire	39	0.3%	6.2 (4.3 to 8.6)	–	–	–
Connecticut	101	0.8%	6.1 (5.0 to 7.5)	43	1.0%	2.5 (1.8 to 3.4)
Rhode Island	28	0.2%	6.0 (4.0 to 8.8)	–	–	–
Vermont	17	0.1%	5.7 (3.2 to 9.3)	–	–	–
Massachusetts	142	1.2%	4.3 (3.6 to 5.1)	54	1.2%	1.5 (1.1 to 2.0)

Boldface indicates statistical significance.

States with fewer than 10 deaths were not included in any calculations for mortality analysis.

a 95% confidence intervals (CIs) were calculated using the Tiwari et al. modification.

b Indiana did not meet the USCS standard for incidence, so their data are not included.

Cervical cancer incidence rates varied widely across states (Table 1). Incidence rates ranged more than 3-fold—from 14.8 per 100 000 in Mississippi (95% CI = 12.5 to 17.4), 14.2 in Louisiana (95% CI = 12.4 to 16.2), and 13.8 in Oklahoma (95% CI = 11.9 to 15.9; all Southeastern/Southwestern states), to 4.3 in Massachusetts (95% CI = 3.6 to 5.1), 5.7 in Vermont (95% CI = 3.2 to 9.3), and 6.0 in Rhode Island (95% CI = 4.0 to 8.8; all in the Northeast). Massachusetts was the closest to reaching the WHO elimination threshold, whereas the rate in Mississippi was 3.7 times higher than the target (Figure 1). Mortality rates showed even greater variation—nearly 5-fold—ranging from 6.8 per 100 000 in Alabama (95% CI = 5.6 to 8.2), 6.3 in Mississippi (95% CI = 4.8 to 8.0), and 5.5 in Oklahoma (95% CI = 4.3 to 7.0) to 1.4 in Wisconsin (95% CI = 1.0 to 2.0), 1.5 in Massachusetts (95% CI = 1.1 to 2.0), and 1.7 in Iowa (95% CI = 1.1 to 2.5) (Table 1).

**Figure 1.**
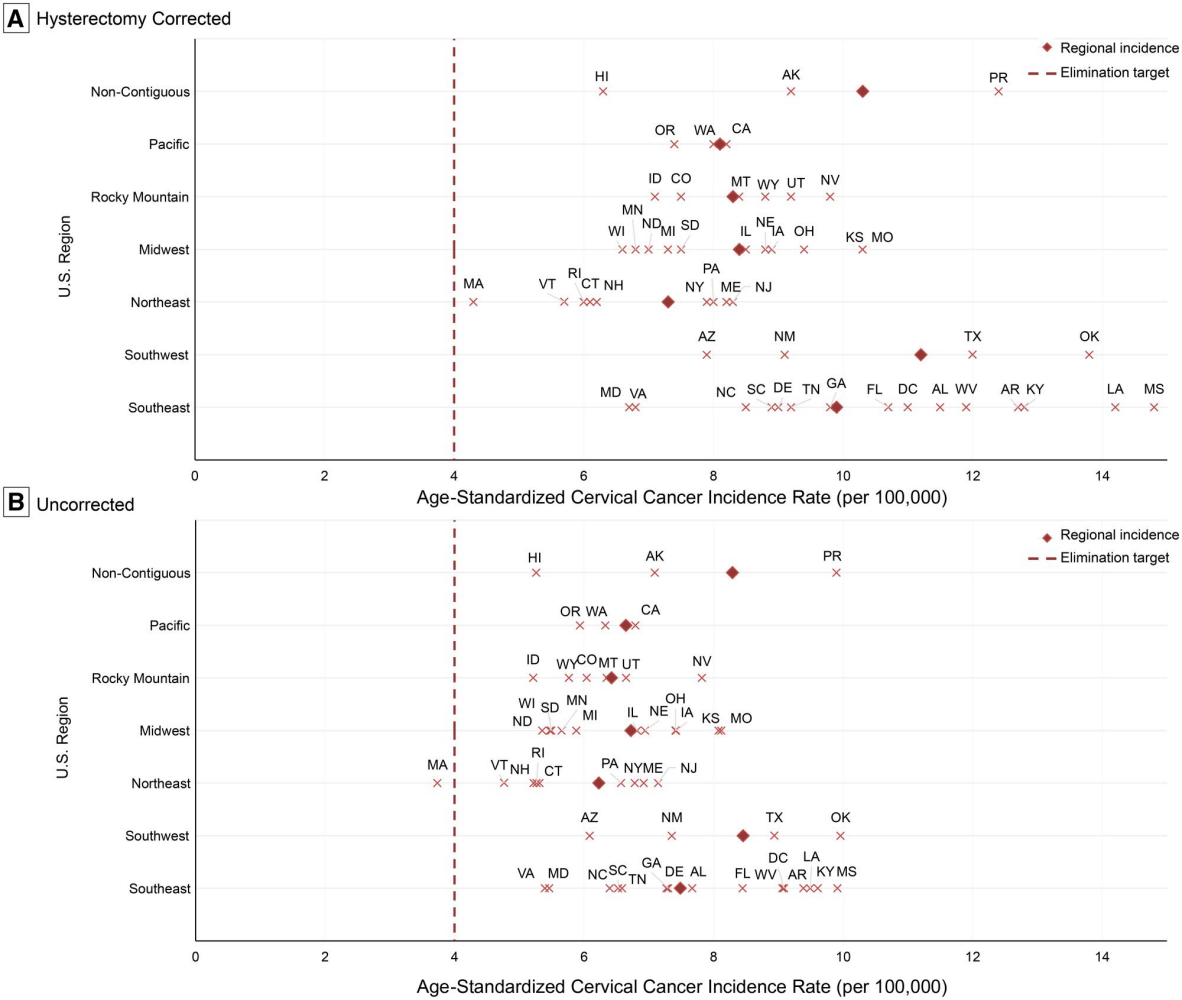
Regional age-standardized cervical cancer incidence rates in the United States benchmarked against WHO elimination goals, 2021. (A). Hysterectomy-corrected. Solid diamond indicates regional incidence rate per 100 000. Vertical dotted line indicates the WHO Elimination Target of 4 cases per 100 000. (B). Uncorrected. Solid diamond indicates regional incidence rate per 100 000. Vertical dotted line indicates the WHO Elimination Target of 4 cases per 100 000.

When comparing the contemporary period of 2017-2021 with the baseline period (2007-2011), a significant decline in cervical cancer incidence (RR = 0.9, 95% CI = 0.90 to 0.94) and mortality (RR = 0.9, 95% CI = 0.87 to 0.93) occurred nationally (Table S1). However, states with the highest absolute cervical cancer incidence rates—Mississippi, Louisiana, Oklahoma—saw no statistically significant change in either incidence or mortality (Figure S1 and Table S1) during these 2 time periods. Incidence RRs in 9 states (mainly from the Midwest), and mortality RRs in 6 states exceeded 1, but the increase was not statistically significant. Notably, in 2023, HPV vaccination coverage (up-to-date on vaccination schedule) in Mississippi was 39.3% (second lowest in the nation) and in Oklahoma it was 49.2% (fourth lowest) (Figure S2). On the other hand, more than 80% of female adolescents were up-to-date with their HPV vaccination schedule in Massachusetts and Rhode Island; the only other state to have achieved this (Healthy People 2030) milestone was Michigan.

Our analysis indicates that, although there has been a modest national decline, substantial regional and state-level variation in cervical cancer incidence and mortality rates persists in the US, with several states showing either no improvement or an increase in these measures. While Massachusetts, Vermont, and Rhode Island are nearing the WHO elimination threshold, states in the Southeast, such as Mississippi and Louisiana, have over 3-fold higher incidence than the threshold. Mortality rates show similarly stark disparities, with the highest mortality rates concentrated in the Southwestern and Southeastern states.

The more than 3-fold variation in hysterectomy-corrected cervical cancer incidence between states with the highest rates (eg, Mississippi) and those with the lowest (eg, Massachusetts) underscores troubling geographic disparities. This concentration of greater incidence and mortality in certain regions may reflect broader issues, including disparities in socioeconomic status, public awareness, preventive service utilization, and access to timely treatment.^16-20^

We further report that incidence and mortality rates have stagnated in most US states. Notably, several recent studies have reported a concerning rise in cervical cancer incidence and mortality rates in low-income counties, rural counties, and regions with persistent poverty, such as Appalachian Kentucky.^16^^,^^17^^,^^21^^,^^22^ These ecological studies have speculated several likely reasons, including reduced access to screening and preventive care, greater disruptions along the screening-to-treatment continuum, use of less effective screening approaches, or increased prevalence of risk factors. Future research is important to understand underlying reasons for these geographic disparities.

Unfortunately, the states with highest cervical cancer incidence, such as Mississippi and Oklahoma, which have not seen significant change in cervical cancer incidence and mortality over the last decade also have some of the lowest HPV vaccination coverage in the nation. On the other hand, states closer to the elimination goal have also achieved or are nearing the Healthy People 2030 HPV vaccination target. Although it is still quite early to realize the definitive population-level impact of the HPV vaccine introduced in 2006—with declines in incidence and mortality thus far limited to younger age groups^23,24^—this cross-sectional assessment indicates that without substantive improvements in HPV vaccination coverage, states with persistently high incidence, mortality, and low vaccination rates, may experience widening disparities as the long-term benefits of vaccination begin to emerge.

Our results should be interpreted in light of a key limitation. The objective of this descriptive study is to document disparities in cervical cancer incidence and mortality and to identify states that may be vulnerable to a future widening of these disparities. Given the limitations of cancer registry data—specifically the lack of information on risk factors (eg, smoking, HPV infection, HIV) and access to timely preventive (HPV vaccination, screening and diagnosis of precancer) and treatment (for precancer and cancer) services—it was not possible to determine the factors contributing to these disparities. Despite this limitation, this study provides a novel descriptive perspective on state variation in cervical cancer incidence and mortality along with progress made and remaining gaps towards elimination.

In conclusion, our findings reveal stark geographic disparities in cervical cancer incidence and mortality rates across the US, with certain states having incidence and mortality rates more than 3 times the WHO elimination target. To truly reach elimination, HPV vaccination, screening and early detection, and timely treatment must be strengthened. Future investigation is critical to identify factors underlying these disparities that could be potentially addressed through targeted prevention efforts to make cervical cancer elimination a reality across all 50 US states, DC, and Puerto Rico.

## Supplementary Material

pkag005_Supplementary_Data

## Data Availability

Data for the National Program of Cancer Registries (NPCR), Surveillance, Epidemiology, and End Results (SEER), Behavioral Risk Factor Surveillance System (BRFSS), and National Immunization Survey (NIS)-Teen Vax View are publicly available at www.cdc.gov/cancer/uscs/public-use, https://seer.cancer.gov/data/, https://www.cdc.gov/brfss/annual_data/annual_data.htm, and https://www.cdc.gov/teenvaxview/interactive/index.html, respectively.
