# Supplementary material for: The cervical cancer divide: state variation in incidence, mortality, and progress toward elimination in the United States
Source: JNCI Cancer Spectr. 2026 Jan 25;10(1):pkag005. doi: 10.1093/jncics/pkag005 (PMC12908680; doi:10.1093/jncics/pkag005)

SUPPLEMENTARY MATERIAL

Table S1: Hysterectomy-Corrected Cervical Cancer Incidence and Mortality Rates by State and Region, 2007-2011 and 2017-2021 (excl. 2020)

	**Age-standardized incidence rate (per 100,000, 95% CI)**			**Age-standardized mortality rate (per 100,000, 95% CI)**		
	Baseline Period (2007-2011)	Contemporary Period (2017-2021)	Rate Ratio	Baseline Period (2007-2011)	Contemporary Period (2017-2021)	Rate Ratio
**US**	10.1 (10.1-10.2)	9.3 (9.2-9.4)	0.9 (0.90-0.94)*	3.5 (3.4-3.5)	3.1 (3.1-3.2)	0.9 (0.87-0.93)*
**Non-Contiguous**	12.9 (12.3-13.6)	11.5 (10.8-12.2)	0.89 (0.79-0.99)*	3.3 (2.9-3.6)	2.8 (2.5-3.2)	0.87 (0.70-1.09)
Puerto Rico	15.5 (14.6-16.4)	14.1 (13.1-15.2)	0.91 (0.80-1.04)	3.6 (3.2-4.1)	3.1 (2.7-3.6)	0.86 (0.65-1.13)
Alaska	8.3 (6.7-10.1)	10.1 (8.2-12.3)	1.22 (0.81-1.84)	3.6 (2.4-5.1)	3.3 (2.2-4.8)	0.92 (0.43-1.95)
Hawaii	8.4 (7.3-9.5)	7.1 (6.1-8.2)	0.85 (0.64-1.12)	2.4 (1.9-3.0)	2.2 (1.7-2.9)	0.93 (0.56-1.54)
**Southwest**	11.7 (11.5-12.0)	11.2 (10.9-11.5)	0.96 (0.91-1.00)	4.2 (4.0-4.4)	4.1 (3.9-4.2)	0.97 (0.89-1.05)
Oklahoma	14.0 (13.1-15.0)	14.0 (13.0-15.0)	1.00 (0.86-1.15)	5.2 (4.6-5.8)	6.2 (5.5-6.9)	1.20 (0.95-1.51)
Texas	12.4 (12.0-12.7)	11.8 (11.5-12.2)	0.96 (0.91-1.01)	4.5 (4.3-4.8)	4.2 (4.0-4.4)	0.93 (0.84-1.02)
New Mexico	9.5 (8.5-10.5)	9.7 (8.6-10.8)	1.02 (0.82-1.27)	3.2 (2.6-3.8)	3.1 (2.6-3.8)	0.99 (0.67-1.46)
Arizona	9.1 (8.5-9.6)	8.0 (7.4-8.5)	0.88 (0.77-1.00)	3.0 (2.7-3.4)	3.1 (2.8-3.5)	1.03 (0.83-1.29)
**Southeast**	11.4 (11.2-11.6)	10.4 (10.2-10.6)	0.91 (0.89-0.94)*	4.3 (4.2-4.4)	3.8 (3.7-3.9)	0.89 (0.85-0.94)*
Mississippi	15.7 (14.5-17.0)	14.2 (13.0-15.4)	0.90 (0.77-1.06)	6.9 (6.1-7.8)	6.3 (5.5-7.1)	0.90 (0.70-1.16)
Louisiana	15.1 (14.2-16.1)	14.1 (13.2-15.1)	0.93 (0.82-1.06)	5.9 (5.3-6.5)	5.5 (4.9-6.1)	0.94 (0.75-1.17)
Arkansas	15.3 (14.2-16.5)	13.2 (12.1-14.4)	0.86 (0.73-1.01)	6.2 (5.5-7.0)	5.5 (4.8-6.3)	0.89 (0.69-1.15)
Kentucky	12.3 (11.5-13.1)	12.9 (12.1-13.8)	1.06 (0.92-1.21)	5.2 (4.7-5.7)	4.3 (3.8-4.8)	0.83 (0.67-1.04)
West Virginia	13.6 (12.4-14.9)	12.7 (11.4-14.1)	0.93 (0.76-1.13)	5.0 (4.2-5.8)	4.3 (3.6-5.1)	0.86 (0.62-1.20)
Alabama	13.7 (12.9-14.6)	12.1 (11.3-13.0)	0.88 (0.77-1.01)	6.0 (5.4-6.6)	5.9 (5.4-6.6)	0.99 (0.81-1.21)
Florida	11.7 (11.4-12.1)	11.0 (10.7-11.4)	0.94 (0.88-1.00)	3.8 (3.6-4.0)	3.8 (3.6-4.0)	0.98 (0.88-1.09)
Georgia	11.8 (11.3-12.4)	10.6 (10.1-11.1)	0.89 (0.81-0.98)*	4.7 (4.4-5.1)	3.6 (3.3-4.0)	0.77 (0.65-0.91)*
South Carolina	11.4 (10.7-12.2)	10.4 (9.7-11.2)	0.92 (0.80-1.05)	4.9 (4.4-5.4)	4.2 (3.7-4.7)	0.86 (0.69-1.07)
Tennessee	12.6 (11.9-13.3)	10.4 (9.8-11.0)	0.82 (0.73-0.93)*	4.9 (4.5-5.4)	4.3 (3.9-4.8)	0.88 (0.72-1.06)
North Carolina	9.7 (9.3-10.2)	9.0 (8.5-9.5)	0.93 (0.84-1.02)	3.5 (3.2-3.8)	3.1 (2.8-3.3)	0.88 (0.74-1.05)
Delaware	11.0 (9.5-12.6)	8.9 (7.6-10.5)	0.81 (0.60-1.10)	3.4 (2.6-4.3)	3.0 (2.3-3.9)	0.90 (0.53-1.52)
District of Columbia	11.6 (9.8-13.8)	8.9 (7.2-10.9)	0.77 (0.53-1.12)	3.3 (2.4-4.6)	3.0 (2.1-4.3)	0.91 (0.46-1.80)
Maryland	8.1 (7.6-8.7)	7.6 (7.0-8.1)	0.93 (0.81-1.06)	3.2 (2.9-3.6)	2.8 (2.5-3.1)	0.87 (0.69-1.08)
Virginia	8.1 (7.7-8.6)	7.2 (6.7-7.6)	0.88 (0.78-0.99)*	3.0 (2.8-3.4)	2.4 (2.2-2.7)	0.80 (0.65-0.98)*

**Midwest**	9.6 (9.4-9.7)	8.9 (8.8-9.1)	0.93 (0.90-0.97)*	3.3 (3.2-3.4)	2.9 (2.8-3.0)	0.90 (0.84-0.96)*
Indiana	10.3 (9.7-10.9)	10.8 (10.1-11.6)	1.05 (0.93-1.20)	3.8 (3.4-4.2)	4.1 (3.7-4.5)	1.08 (0.88-1.31)
Kansas	10.1 (9.2-11.1)	10.7 (9.7-11.8)	1.06 (0.88-1.28)	3.0 (2.5-3.5)	3.8 (3.3-4.5)	1.28 (0.92-1.79)
Missouri	10.9 (10.3-11.5)	10.7 (10.1-11.4)	0.98 (0.87-1.11)	3.9 (3.6-4.3)	3.6 (3.2-4.0)	0.91 (0.74-1.12)
Nebraska	9.2 (8.2-10.3)	9.9 (8.8-11.1)	1.07 (0.85-1.35)	2.8 (2.2-3.4)	3.0 (2.4-3.7)	1.10 (0.72-1.69)
Ohio	10.0 (9.6-10.4)	9.7 (9.3-10.2)	0.97 (0.89-1.06)	3.9 (3.7-4.2)	3.3 (3.1-3.6)	0.84 (0.72-0.97)*
Iowa	8.4 (7.7-9.2)	9.4 (8.6-10.4)	1.12 (0.93-1.35)	2.9 (2.5-3.4)	2.1 (1.7-2.5)	0.71 (0.50-1.00)
Illinois	10.7 (10.3-11.1)	8.8 (8.5-9.3)	0.83 (0.76-0.90)*	3.7 (3.5-4.0)	2.9 (2.7-3.1)	0.78 (0.67-0.90)*
Michigan	9.5 (9.1-10.0)	8.1 (7.6-8.5)	0.84 (0.76-0.94)*	3.0 (2.8-3.3)	2.9 (2.6-3.1)	0.96 (0.80-1.14)
South Dakota	8.5 (7.0-10.1)	8.0 (6.5-9.7)	0.94 (0.64-1.38)	3.2 (2.3-4.3)	3.3 (2.4-4.4)	1.03 (0.55-1.90)
North Dakota	7.9 (6.4-9.7)	7.6 (6.0-9.4)	0.96 (0.63-1.48)	2.1 (1.4-3.1)	2.2 (1.4-3.2)	1.02 (0.44-2.36)
Wisconsin	7.3 (6.9-7.9)	7.1 (6.6-7.7)	0.97 (0.83-1.12)	2.2 (1.9-2.5)	1.9 (1.6-2.1)	0.84 (0.64-1.11)
Minnesota	7.3 (6.8-7.8)	6.7 (6.1-7.2)	0.92 (0.79-1.07)	1.9 (1.7-2.2)	1.7 (1.5-2.0)	0.89 (0.66-1.20)
**Pacific**	9.4 (9.2-9.6)	8.5 (8.3-8.7)	0.90 (0.86-0.94)*	3.1 (3.0-3.2)	2.8 (2.7-2.9)	0.90 (0.84-0.98)*
California	9.6 (9.4-9.9)	8.6 (8.4-8.8)	0.89 (0.85-0.94)*	3.2 (3.0-3.3)	2.9 (2.8-3.0)	0.92 (0.84-1.00)
Oregon	8.9 (8.3-9.7)	8.3 (7.6-9.0)	0.93 (0.79-1.09)	3.0 (2.6-3.4)	2.7 (2.3-3.1)	0.89 (0.67-1.19)
Washington	8.6 (8.1-9.1)	8.2 (7.7-8.7)	0.95 (0.84-1.08)	2.9 (2.6-3.2)	2.4 (2.1-2.7)	0.84 (0.67-1.06)
**Rocky Mountain**	8.5 (8.2-8.9)	8.5 (8.2-8.9)	1.00 (0.91-1.10)	2.8 (2.6-3.1)	2.8 (2.6-3.0)	0.98 (0.83-1.16)
Wyoming	12.4 (10.2-15.0)	12.5 (10.1-15.3)	1.00 (0.67-1.49)	4.8 (3.4-6.6)	3.8 (2.5-5.5)	0.78 (0.38-1.62)
Nevada	9.7 (8.9-10.7)	10.1 (9.2-11.0)	1.03 (0.86-1.24)	3.4 (2.8-4.0)	3.9 (3.3-4.5)	1.16 (0.84-1.60)
Idaho	8.8 (7.7-10.1)	9.7 (8.5-11.0)	1.10 (0.84-1.44)	3.8 (3.0-4.7)	2.7 (2.1-3.5)	0.72 (0.44-1.16)
Utah	7.5 (6.7-8.5)	8.0 (7.1-8.9)	1.06 (0.84-1.34)	2.0 (1.5-2.6)	2.9 (2.4-3.6)	1.45 (0.92-2.30)
Colorado	7.8 (7.2-8.4)	7.6 (7.0-8.2)	0.97 (0.83-1.13)	2.5 (2.1-2.9)	2.2 (1.9-2.6)	0.89 (0.67-1.19)
Montana	8.6 (7.3-10.1)	7.6 (6.4-9.1)	0.89 (0.63-1.24)	2.4 (1.7-3.3)	2.1 (1.5-3.0)	0.87 (0.44-1.71)
**Northeast**	8.9 (8.7-9.1)	7.7 (7.5-7.9)	0.87 (0.83-0.90)*	2.7 (2.6-2.8)	2.2 (2.1-2.3)	0.80 (0.75-0.87)*
New York	9.6 (9.3-9.9)	8.4 (8.1-8.7)	0.87 (0.81-0.93)*	3.0 (2.9-3.2)	2.3 (2.2-2.5)	0.77 (0.68-0.87)*
Pennsylvania	9.8 (9.4-10.2)	8.4 (8.0-8.8)	0.86 (0.79-0.93)*	3.0 (2.8-3.2)	2.5 (2.3-2.8)	0.85 (0.73-0.99)*
New Jersey	9.7 (9.3-10.2)	8.2 (7.8-8.7)	0.85 (0.77-0.94)*	3.0 (2.7-3.2)	2.4 (2.2-2.7)	0.81 (0.68-0.97)*
Rhode Island	7.7 (6.6-8.9)	7.4 (6.2-8.8)	0.97 (0.70-1.34)	1.9 (1.4-2.6)	1.7 (1.1-2.4)	0.87 (0.44-1.69)
Maine	8.2 (7.2-9.4)	6.9 (5.9-8.1)	0.84 (0.63-1.12)	2.4 (1.9-3.0)	1.8 (1.3-2.4)	0.73 (0.42-1.26)
New Hampshire	6.3 (5.4-7.3)	6.3 (5.3-7.4)	0.99 (0.72-1.36)	2.4 (1.9-3.1)	1.8 (1.3-2.4)	0.74 (0.43-1.28)
Connecticut	7.4 (6.8-8.0)	6.0 (5.4-6.7)	0.82 (0.68-0.98)*	2.1 (1.8-2.4)	1.8 (1.5-2.1)	0.86 (0.61-1.20)

Vermont	5.2 (4.1-6.6)	6.0 (4.7-7.6)	1.15 (0.71-1.87)	1.7 (1.1-2.5)	1.8 (1.1-2.8)	1.05 (0.42-2.57)
Massachusetts	6.1 (5.7-6.6)	5.5 (5.1-5.9)	0.89 (0.77-1.02)	1.8 (1.6-2.0)	1.4 (1.2-1.6)	0.80 (0.61-1.04)

Footnotes:

*Denotes a statistically significant rate ratio

Figure S1. Maps of Hysterectomy-Corrected Age-Standardized Cervical Cancer Incidence and Mortality Rates by State, 2007-2011 and 2017-2021 (excl. 2020)

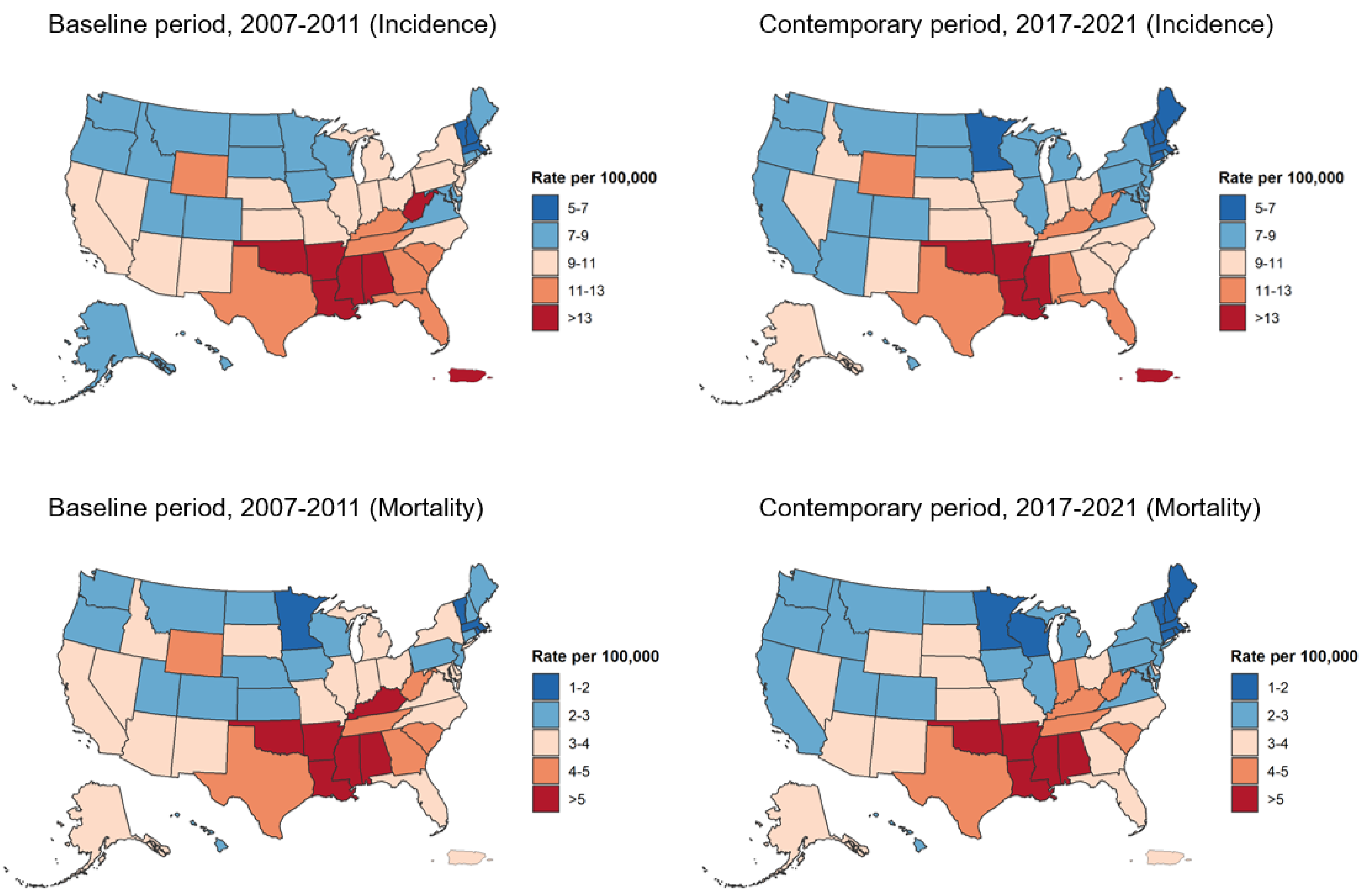

Figure S2. Human Papillomavirus (HPV) Vaccine Coverage among Females Aged 13-17 in the United States, 2023.

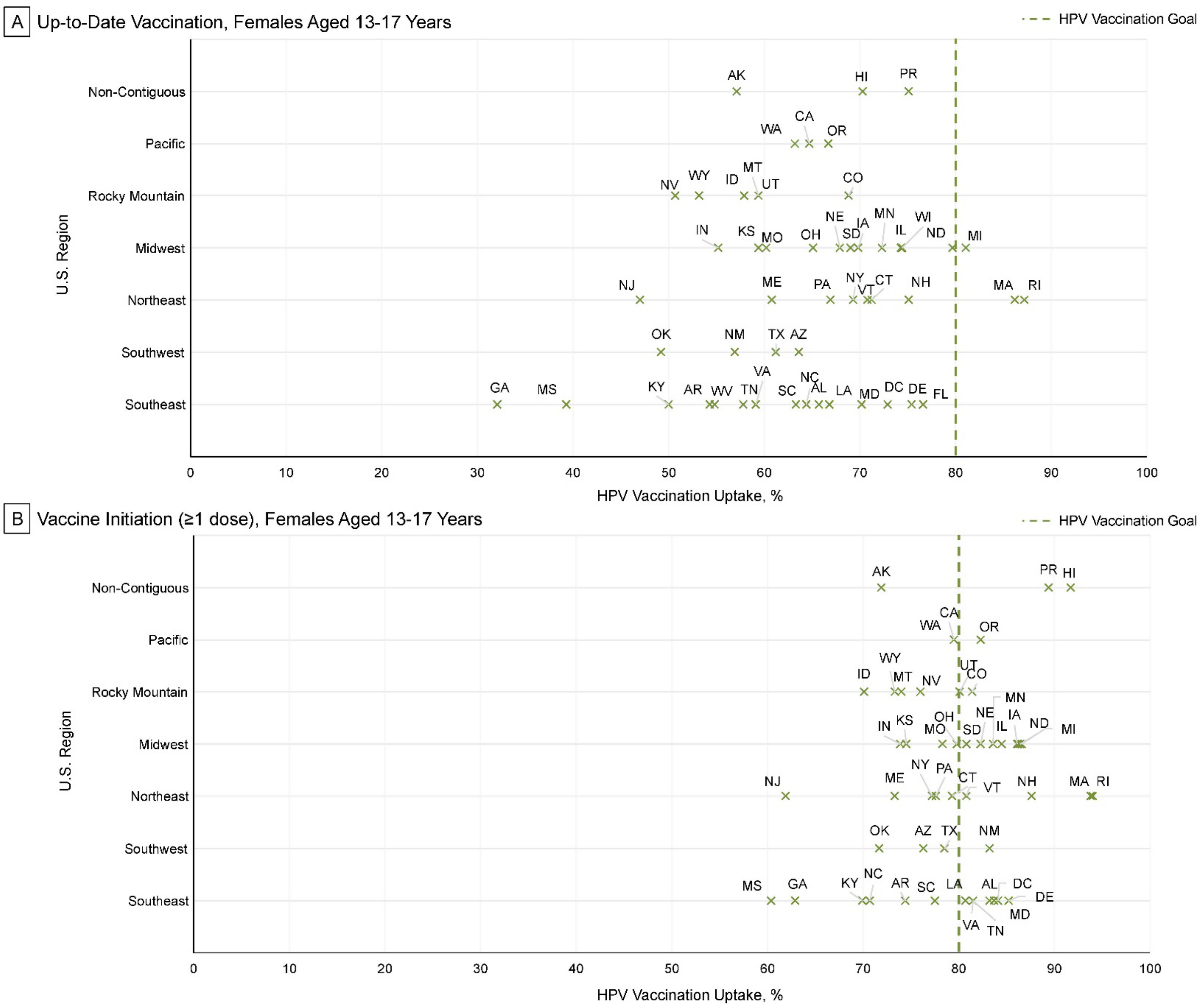

Supplement: pkag005_Supplementary_Data [file pkag005_supplementary_data.pdf]
